# Trends in Clinical Indications for Plasma Exchange in Adult Patients Between 2008 and 2024 in Bogotá, Colombia

**DOI:** 10.1002/jca.70161

**Published:** 2026-07-28

**Authors:** Edgar Julián Reyes, Federico Tovar‐Avendaño, Camilo Alberto Gonzalez‐Gonzalez, Diana Carolina Vargas‐Angel, Jorge Pulido, Paula Violeta Rodríguez, Deiner‐Yivelson Muñoz‐Delgado, Kateir Contreras‐Villamizar

**Affiliations:** ^1^ Departamento de Medicina Interna, Unidad de Nefrología Hospital Universitario San Ignacio Bogotá Colombia; ^2^ Facultad de Medicina, Departamento de Medicina Interna Pontificia Universidad Javeriana Bogotá Colombia; ^3^ Facultad de Medicina, Departamento de Medicina Interna Universidad Nacional de Colombia Bogotá Colombia; ^4^ Departamento de Medicina Interna Hospital Universitario Nacional de Colombia Bogotá Colombia; ^5^ Unidad Renal Grupo Keralty, Clínica Colsanitas Bogotá Colombia; ^6^ Departamento de Medicina Interna, Unidad de Nefrología Hospital Universitario Clinica San Rafael Bogotá Colombia

**Keywords:** ANCA‐associated vasculitis, apheresis, ASFA guidelines, autoimmune diseases, immune‐mediated neurological disorders, Latin America, neuromyelitis optica spectrum disorders

## Abstract

This multicenter retrospective study aimed to describe temporal trends in clinical indications for plasma exchange (PE) in adult patients at four quaternary‐care hospitals in Bogotá (2008–2024) and to examine their distribution across successive ASFA guideline editions. Adults ≥ 18 years who received ≥ 1 PE session were included; indications were coded using ICD‐10, and outcomes were classified as complete improvement, partial improvement, no change, or death. Descriptive statistics were performed using RStudio. Of 970 patients screened, 758 met eligibility criteria. Median age was 47 years (IQR 33–60); 62.4% were women. Neurological and rheumatological indications predominated (*n* = 634, 83.6%), particularly neuromyelitis optica spectrum disorders and ANCA‐associated vasculitis. A total of 3954 sessions were performed (median: 5 per patient; IQR: 5–6). Hypocalcemia was the most frequent complication. Overall, 74.0% achieved complete or partial improvement, with higher rates in neurological conditions. Sixty‐nine deaths occurred (9.1%), mostly attributed to systemic autoimmune diseases. Over the study period, neuroimmunological indications increased markedly, while use of PE for ANCA‐associated vasculitis declined following its reclassification to ASFA category III in 2023. ASFA categories I and II accounted for 92.7% of indications; Category I representation declined from 66.7% (2008–2010) to 40.6% (2023–2024), while case volume grew from 21 to 266 patients per triennium. Over 16 years, PE indications shifted substantially toward immune‐mediated neurological disorders, driven by expanded diagnostic availability for AQP4‐IgG and MOG‐IgG and evolving ASFA recommendations. These findings underscore the need for prospective studies and a national PE registry in Colombia.

## 1 Introduction

Plasma exchange (PE) is an extracorporeal procedure whose primary objective is the removal of antibodies, immune complexes, cytokines, and other pathological macromolecules from the plasma [1]. In addition to its depurative effect, an immunomodulatory role in T lymphocytes has been proposed, promoting a shift from a Th1 to a Th2 phenotype, with suppression of IL‐2 and a reduction in IFN‐γ [2]. Through these mechanisms, PE has become an important therapeutic tool across multiple medical specialties [3].

Since its clinical expansion in the 1970s, particularly in autoimmune diseases such as systemic lupus erythematosus, Goodpasture syndrome, and myasthenia gravis [4], PE has undergone progressive development. The establishment of the American Society for Apheresis (ASFA) in 1982 and the publication of its first guideline in 1986 [4, 5], enabled the standardization of indications and the periodic updating of available evidence. This process has led to a substantial increase in the number of included conditions, from 61 in the first edition to 166 in the 2023 version, although with a high proportion of category III indications supported by limited evidence [6].

Despite these advances, significant challenges remain, particularly in developing countries, where access to PE is restricted by the low frequency of certain conditions, the scarcity of robust evidence, the lack of therapeutic alternatives for potentially life‐threatening diseases, and logistical and economic constraints. In Latin America, available information remains fragmented and is derived mainly from single‐center series with small sample sizes, a focus on specific pathologies, and short follow‐up periods [7, 8, 9, 10, 11, 12, 13, 14, 15, 16].

In this context, a broader, more standardized characterization of real‐world use of PE in the region is needed. Therefore, the objective of this study was to describe trends in the clinical indications for PE among adult patients treated at four quaternary‐care hospitals in Bogotá between 2008 and 2024, and to analyze their distribution across successive editions of the ASFA guidelines.

## 2 Materials and Methods

A multicenter retrospective study was conducted, including patients older than 18 years treated in the nephrology departments of four high‐complexity institutions in Bogotá. All patients who received at least one PE session between January 1, 2008, and December 31, 2024, were considered eligible. Patients with incomplete clinical records or without documented information regarding diagnosis or indication for PE were excluded.

The protocol was approved by the Institutional Review Boards and Ethics Committees of all participating institutions: the Research and Ethics Committee of Hospital Universitario San Ignacio and Pontificia Universidad Javeriana on December 19, 2024 (codes FM‐CIE‐1408‐24 and 2024/289), the Hospital Universitario Nacional de Colombia (code CEI‐2024‐12‐07), the Fundación Universitaria Sanitas (code CEIFUS 866‐25), and the Hospital Universitario Clínica San Rafael (code CEI 054‐2025). The study was conducted in accordance with the ethical principles of the Declaration of Helsinki of 1964 and its subsequent amendments. All committees waived individual informed consent, as this retrospective review did not include identifiable patient information or personal images, thereby ensuring strict data confidentiality.

The initial case identification was performed using each institution's administrative databases, employing the Colombian Unique Classification of Health Procedures (CUPS) code 911302, which corresponds to PE. Subsequently, clinical data were extracted from the respective electronic health record systems. Prior to data collection, an investigator's manual was developed to establish standardized guidelines and procedures for data abstraction.

Data were recorded and managed using the Research Electronic Data Capture (REDCap) [17] platform of Hospital Universitario San Ignacio. Each participating institution had independent, restricted access and could not view records from other centers, ensuring data confidentiality and security. Data consistency was verified, and duplicate records identified within each institution or across institutions during the study period were removed as appropriate.

Indications for therapy were recorded using the corresponding ICD‐10 code [18] for each condition, to ensure uniform data collection. Data on immunosuppressive therapy and clinical outcomes were collected exclusively from the index hospitalization during which PE was performed. Clinical outcomes were assessed according to the treating specialist's clinical judgment, using the disease‐specific clinical criteria and assessment scales routinely applied for each indication. Outcomes were classified as complete improvement (full recovery of organ involvement), partial improvement (incomplete recovery), no change (no documented clinical improvement), or death (in‐hospital mortality). Complications related to both vascular access and the procedure were also documented. Finally, information regarding the prescription of the first PE session for each patient was collected. All procedures were performed using membrane‐based PE with hollow‐fiber plasma separators (Plasmaflow 0.8, Plasmaflow 0.9, Plasmart 700, or ST 2000). Recorded prescription parameters included vascular access type, blood flow rate (Qb), total volume exchanged, estimated plasma volume (calculated as body weight [kg] × 70 × [1−hematocrit/100]), replacement fluid composition, and anticoagulation method. When anticoagulation was required, systemic heparin was used; citrate anticoagulation was not employed at any participating institution.

A descriptive statistical analysis was performed to summarize baseline characteristics. The distribution of continuous variables was assessed visually using histograms and Q–Q plots. Variables showing non‐normal distributions are presented as median and interquartile range (IQR); normally distributed variables are presented as mean ± standard deviation. Categorical variables were presented as absolute frequencies and percentages.

Descriptive tables were generated for indications, prescription characteristics, and therapy‐related complications. Proportion plots by triennia were constructed to illustrate the most frequent indications for PE and their classification according to ASFA guidelines, using the publication dates of the different guideline editions as cut‐off points. Data analysis was performed using RStudio software, version 4.4.2 [19].

## 3 Results

A total of 970 patients were identified from the administrative databases of the participating institutions. After applying eligibility criteria and removing duplicate records within and across institutions, 758 patients were included in the final analysis. Their general characteristics are shown in Table 1. The median age was 47 years (IQR 33–60), and the majority were women (62.4%).

**TABLE 1:** Baseline characteristics of 758 adult patients who received plasma exchange at four high‐complexity centers in Bogotá, Colombia (2008–2024).

Variable	Value
Number of patients (*N*)	758
Age, years, median (IQR)	47.0 (33.0–60.0)
Female, *n* (%)	473 (62.4%)
Weight, kg, median (IQR)	65.0 (58.0–72.0)
Hematocrit^a^, %, median (IQR)	40.0 (30.1–44.8)
Platelet count^b^, mean (SD)	245 019 (±113 335)
Hospital location^c^, *n* (%)	
General ward	342 (45.1%)
Intensive Care Unit	207 (27.3%)
Intermediate Care Unit	87 (11.5%)
Emergency Department	57 (7.5%)
Resuscitation Room	17 (2.2%)
Procedure Room	9 (1.2%)
Hemodialysis unit	33 (4.4%)
Indication for plasma exchange by system, *n* (%)	
Neurological	504 (66.5%)
Rheumatological	130 (17.2%)
Hematological	56 (7.4%)
Renal	48 (6.3%)
Endocrinological	16 (2.1%)
Cardiac	4 (0.5%)
ASFA Category	
Category I	321 (42.3%)
Category II	382 (50.4%)
Category III	51 (6.7%)
Category IV	4 (0.5%)
Hospital length of stay, days, median (IQR)	16.0 (12.0–25.0)

Abbreviations: ASFA, American Society for Apheresis; Category I, first‐line therapy; Category II, second‐line therapy; Category III, limited evidence/not routinely recommended; Category IV, ineffective therapy or potentially harmful; SD, standard deviation.

^a^ Hematocrit from the first session of plasma exchange.

^b^ Platelet count from the first session of plasma exchange.

^c^ Hospital location at the time the therapy was performed. Other settings or missing data are not shown (*n* = 6).

Approximately 45% of patients initiated PE in general ward settings, whereas 39% received treatment in critical care units (intensive care units—ICUs or intermediate care units). The distribution by clinical setting varied markedly by indication: NMO spectrum disorders and renal transplant rejection were managed predominantly in general wards (93.9% and 94.7%, respectively), whereas conditions such as catastrophic antiphospholipid syndrome (90%), severe systemic lupus erythematosus (79%), myasthenia gravis (64%), Guillain–Barré syndrome (62%), and ANCA‐associated vasculitis (44%) were more frequently treated in critical care settings.

Neurological and rheumatological indications accounted for the majority of cases (*n* = 634). Among neurological indications, neuromyelitis optica spectrum disorders and other neuroinflammatory conditions such as acute disseminated encephalomyelitis and Guillain–Barré syndrome were most prominent. Within rheumatological indications, the most frequent conditions were ANCA‐associated vasculitis and severe manifestations of systemic lupus erythematosus, mainly presenting as pulmonary–renal syndrome and diffuse alveolar hemorrhage, respectively. Further details are provided in Table S1.

Among hematological indications, thrombotic microangiopathies ranked seventh in frequency (*n* = 38). Within this group, thrombotic thrombocytopenic purpura was the most relevant condition (*n* = 20), with 14 cases confirmed by ADAMTS‐13 activity levels, while six remained clinical suspicions due to patient death prior to diagnostic confirmation.

Regarding renal indications, the majority corresponded to acute antibody‐mediated rejection (*n* = 38). Additionally, two cases of post‐transplant focal segmental glomerulosclerosis relapse were recorded; these patients initially received seven inpatient PE sessions and subsequently continued outpatient therapy, with one completing a total of 33 sessions. In the ANCA‐associated vasculitis group, 21 patients received PE due to rapidly progressive glomerulonephritis.

A median of 5 (IQR 5–6) PE sessions were performed per patient. Detailed prescription characteristics are presented in Table 2. Temporary right internal jugular catheters were the most frequently used vascular access, with a low rate of access‐related complications; uncomplicated arterial puncture was the most common event, accounting for 72.2% of all access‐related complications. The median exchanged plasma volume was 3000 mL (IQR 2550–3600), and 5% albumin was the most commonly used replacement fluid, accounting for 65.6% of all procedures and 69.3% after excluding TTP/HUS cases. Circuit anticoagulation was not required in 97.9% of sessions. The most frequent procedure‐related complication was hypocalcemia, occurring in 77.2% of patients who experienced any complication. In all cases, hypocalcemia was managed with intravenous calcium gluconate administered only after documented hypocalcemia, without routine prophylactic supplementation.

**TABLE 2:** Prescription characteristics of the first plasma exchange session (*N* = 758).

Variable	Value
Total number of sessions, *n*	3954
Number of sessions, median (IQR)	5 (IQR 5–6)
Vascular access type, *n* (%)	
Right internal jugular temporary catheter	548 (72.3)
Left internal jugular temporary catheter	44 (5.8)
Right femoral temporary catheter	126 (16.6)
Left femoral temporary catheter	13 (1.7)
Right internal jugular tunneled catheter	8 (1.1)
Right femoral tunneled catheter	1 (0.1)
Arteriovenous fistula	18 (2.4)
Vascular access complication, *n* (%)	
Yes	54 (7.1%)
Procedure complication type, *n* (%)	
Arterial puncture	39 (72.2)
Hematoma	6 (11.1)
Catheter‐associated infection	3 (5.6%)
Pneumothorax	2 (3.7)
Hemothorax	1 (1.9)
Blood flow rate (Qb), mL/min, mean (SD)	162.5 (±27.8)
Plasma volume exchanged, mL, median (IQR)	3000 (2550–3600)
Plasma volume ratio (exchanged/estimated), median (IQR)^a^	1.1 (0.9–1.2)
Replacement fluid	
Albumin 5%	497 (65.6)
Colloid	78 (10.3)
Fresh frozen plasma	166 (21.9)
Mixed^b^	8 (1.1)
Amount of albumin, g, mean (SD)	149.6 (±44.9)
Filter type, *n* (%)	
Plasmaflow 0.8	339 (44.7)
Plasmart 700	231 (30.5)
ST 2000	103 (13.6)
Plasmaflow 0.9	1 (0.1)
Anticoagulation, *n* (% of sessions)	
No	3741 (97.9)
Heparin	79 (2.1)
Complication related to plasma exchange, *n* (%)	
Yes	145 (19.1)
Type of plasma exchange complication	
Hypocalcemia	112 (77.2)
Hypomagnesemia	71 (49.0)
Symptomatic hypotension	3 (2.1)
Minor allergic reaction	3 (2.1)
Minor bleeding^c^	1 (0.7)
Infection other than bacteremia	1 (0.7)
Clinical outcome	
Complete improvement	285 (37.6)
Partial improvement	274 (36.1)
No change	118 (15.6)
Death	69 (9.1)
Worsening	1 (0.1)

Abbreviations: FFP, fresh frozen plasma; g, grams; Qb, blood flow rate; SD, standard deviation.

^a^ Plasma volume to be exchanged during the first session of plasma exchange.

^b^ Combination of albumin with fresh frozen plasma.

^c^ Bleeding not requiring transfusion.

At the end of treatment, approximately 74% of patients showed complete or partial clinical improvement, predominantly among neurological conditions (78.2%). In contrast, only 56.2% of patients with systemic autoimmune diseases achieved some degree of improvement.

**FIGURE 1:**
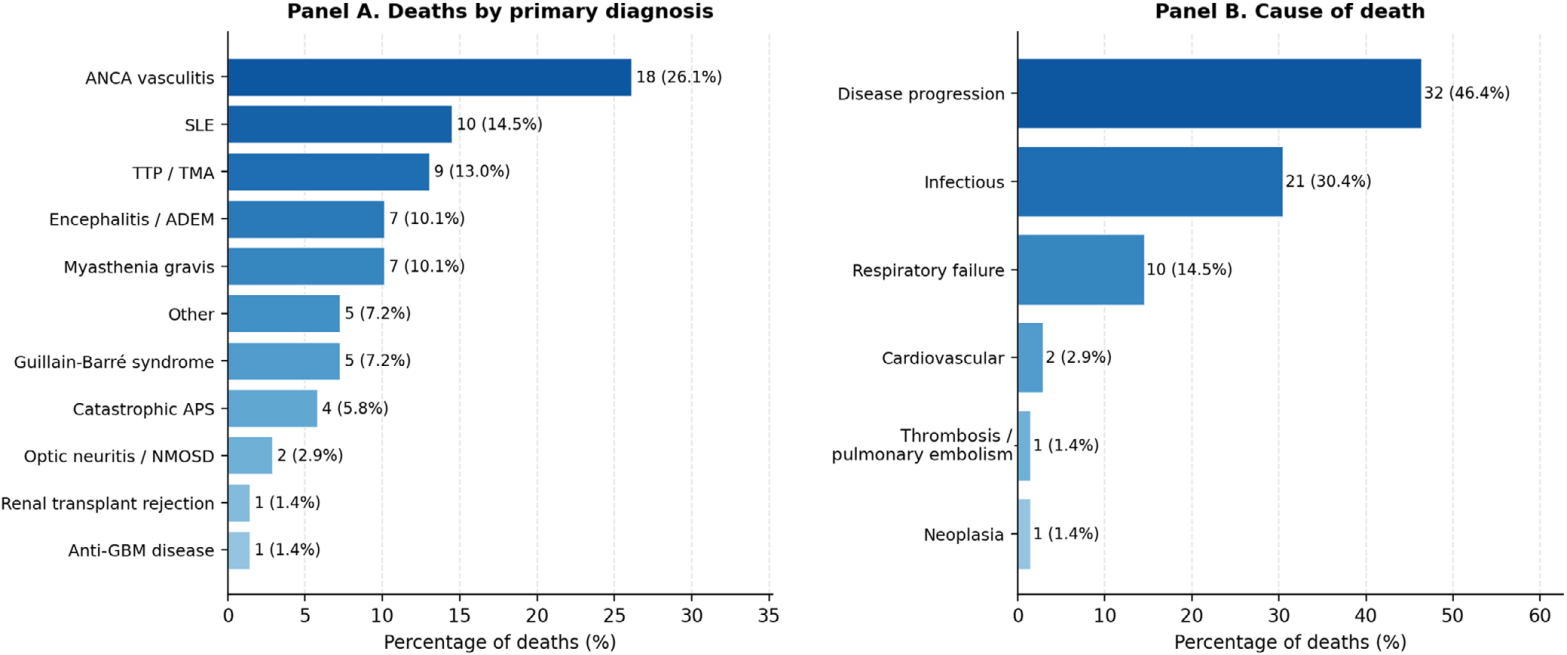
In‐hospital mortality by diagnosis and cause of death (*N* = 69 deaths). (A) Distribution of deaths by primary diagnosis (ICD‐10 code). (B) Distribution by cause of death. ANCA, antineutrophil cytoplasmic antibody; Anti‐NMDAR, anti‐N‐methyl‐D‐aspartate receptor; APS, antiphospholipid syndrome; NMOSD, neuromyelitis optica spectrum disorder; SLE, systemic lupus erythematosus; TTP, thrombotic thrombocytopenic purpura.

A total of 69 deaths were recorded, most of them associated with autoimmune conditions, including ANCA‐associated vasculitis (26.1%), systemic lupus erythematosus (14.5%), and catastrophic antiphospholipid syndrome (5.8%). Rheumatological and hematological indications were associated with the highest in‐group mortality rates (25.4% and 25.0%, respectively), compared to neurological disorders (4.2%) (Figure 1A). At the individual diagnosis level, catastrophic antiphospholipid syndrome (40.0%), systemic lupus erythematosus (25.0%), ANCA‐associated vasculitis (23.7%), and thrombotic microangiopathy (23.7%) exhibited the highest case‐fatality rates. The leading causes of death were progression of the underlying disease (46.4%) and infectious complications (30.4%), followed by respiratory failure (14.5%); no death was attributable to a direct complication of the procedure (Figure 1B). Notably, 31.9% of deaths occurred during active PE treatment, defined as death on the same day as the last session (D1: 13.0%) or within 48 h of the last session in patients who did not complete a standard five‐session cycle (D2: 23.2%).

Concomitant immunosuppressive therapy was documented in 651 patients (85.9%), while 107 (14.1%) received PE without any immunosuppressive agent. The five most frequently used immunosuppressive drugs were methylprednisolone (516 patients, 68.1%), prednisolone (356, 47.0%), cyclophosphamide (108, 14.2%), azathioprine (72, 9.5%), and rituximab (50, 6.6%). The highest rates of immunosuppressive co‐treatment were observed in neuromyelitis optica spectrum disorder (97.0%), ANCA‐associated vasculitis (98.7%), and systemic lupus erythematosus with renal involvement (97.1%), whereas Guillain–Barré syndrome showed the lowest co‐treatment rate among the most frequent indications (27.7%).

**FIGURE 2:**
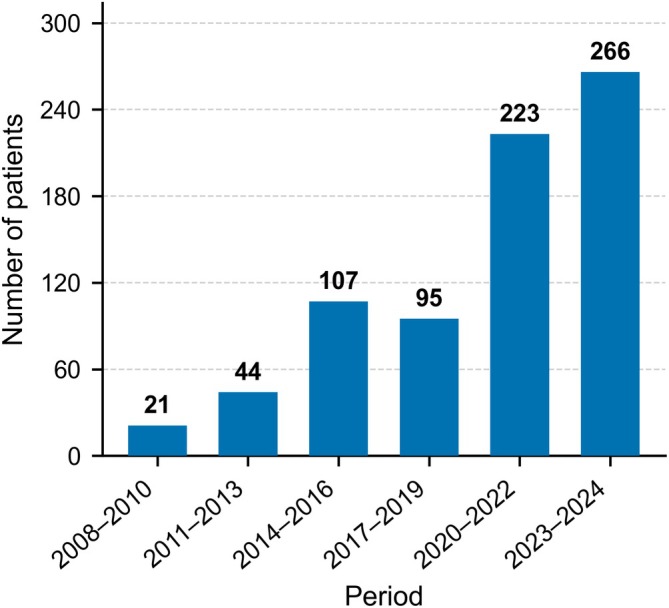
Number of patients who received plasma exchange per triennium (2008–2024). Case volume increased progressively from 21 patients in 2008–2010 to 266 in 2023–2024.

**FIGURE 3:**
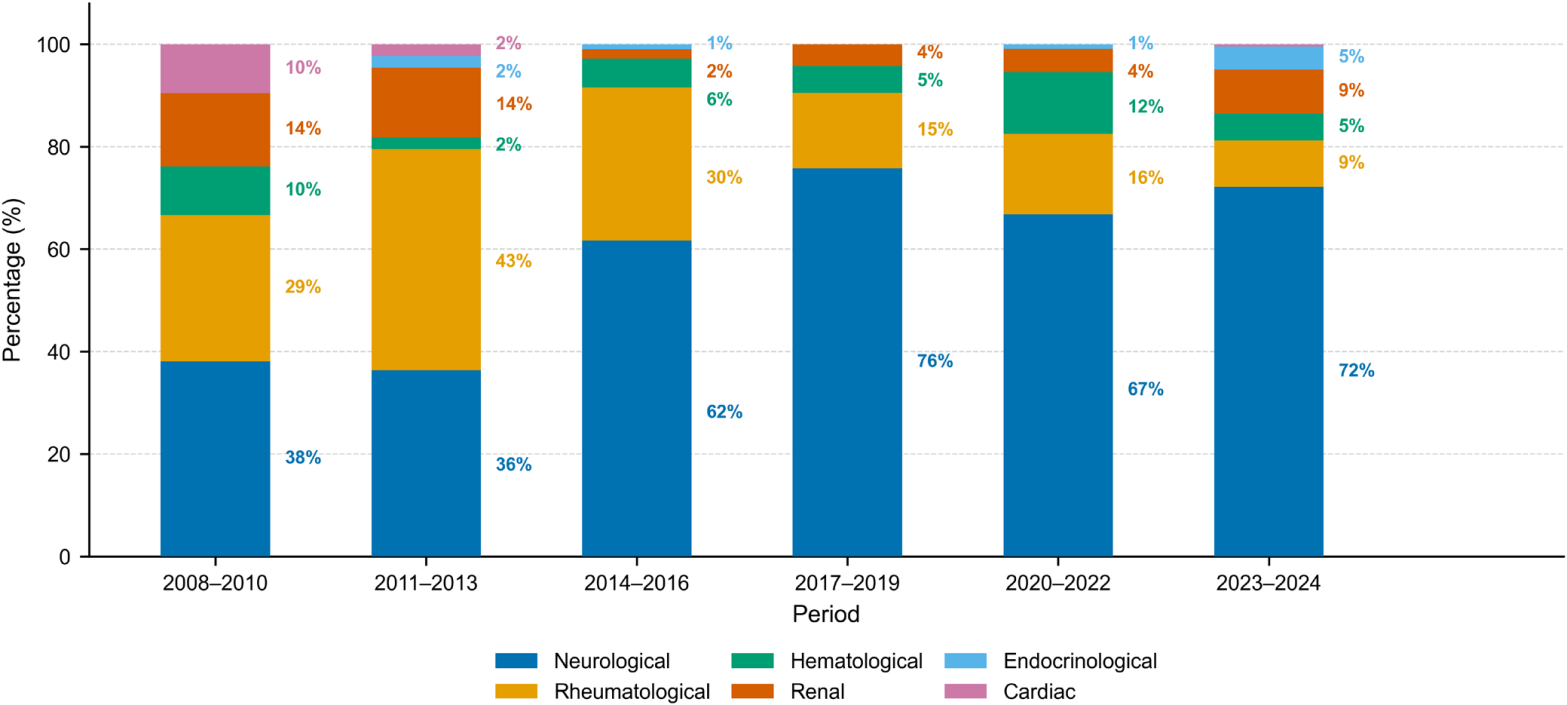
Distribution of plasma exchange indications by organ system per triennium (2008–2024), expressed as percentage of cases in each period. Neurological indications rose from 38% in 2008–2010 to 72% in 2023–2024.

**FIGURE 4:**
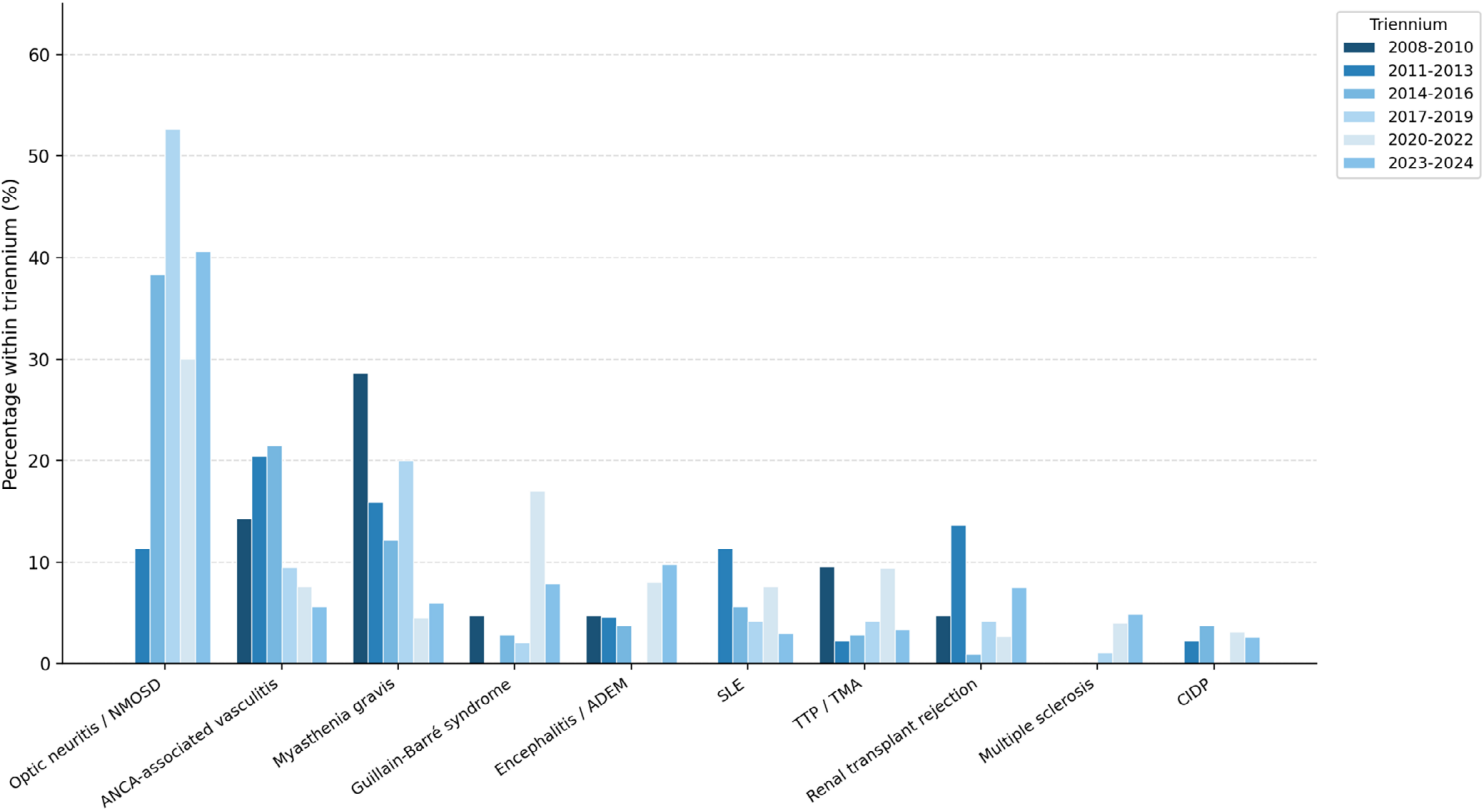
Top 10 indications for plasma exchange by triennium (2008–2024), expressed as percentage of cases within each triennium. The supplementary numerical data are provided in Table S1. ADEM, acute disseminated encephalomyelitis; ANCA, anti‐neutrophil cytoplasmic antibody; APS, antiphospholipid syndrome; GBS, Guillain–Barré syndrome; NMOSD, neuromyelitis optica spectrum disorder; SLE, systemic lupus erythematosus with renal involvement; TTP, thrombotic thrombocytopenic purpura.

**FIGURE 5:**
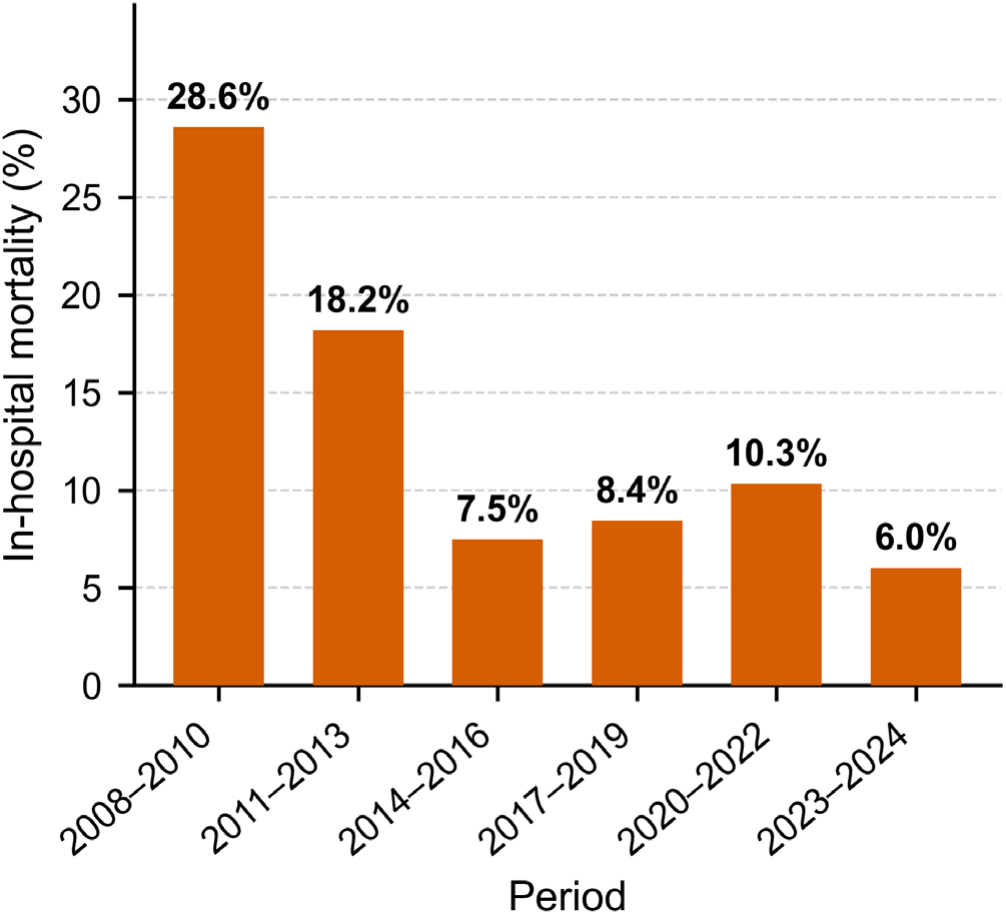
In‐hospital mortality rate per triennium (2008–2024), expressed as percentage of patients who died during each period. Mortality declined from 28.6% in 2008–2010 to 6.0% in 2023–2024.

Over the study period, a progressive change in PE indications was observed, accompanied by a substantial increase in case volume—from 21 cases in 2008–2010 to 266 in 2023–2024, with nearly two‐thirds of the cohort concentrated in the final two triennia (Figure 2). In the early triennia (2008–2013), systemic autoimmune conditions predominated: rheumatological indications accounted for up to 43% of cases, ASFA Category I indications represented the majority (59%–67%), and the leading diagnoses were ANCA‐associated vasculitis, myasthenia gravis, and renal allograft rejection. From 2014 onward, neuromyelitis optica spectrum disorders—including optic neuritis—became the predominant indication, with frequencies ranging from 29% to 53% across subsequent triennia. Concurrently, neurological indications rose from 38% in 2008–2010 to 72% in 2023–2024, while rheumatological indications declined from 43% to 9% (Figure 3). This diagnostic shift was accompanied by a progressive increase in ASFA Category II indications, which peaked at 61% in 2017–2019, and by the emergence of other neuroinflammatory conditions such as Guillain–Barré syndrome and acute encephalitis as leading diagnoses. The distribution of the 10 most frequent indications across triennia is shown in Figure 4 and Table S1. In‐hospital mortality declined from 28.6% in the earliest triennium to 6.0% in 2023–2024, with a transient increase observed during 2020–2022 (10.3%) (Figure 5).

**FIGURE 6:**
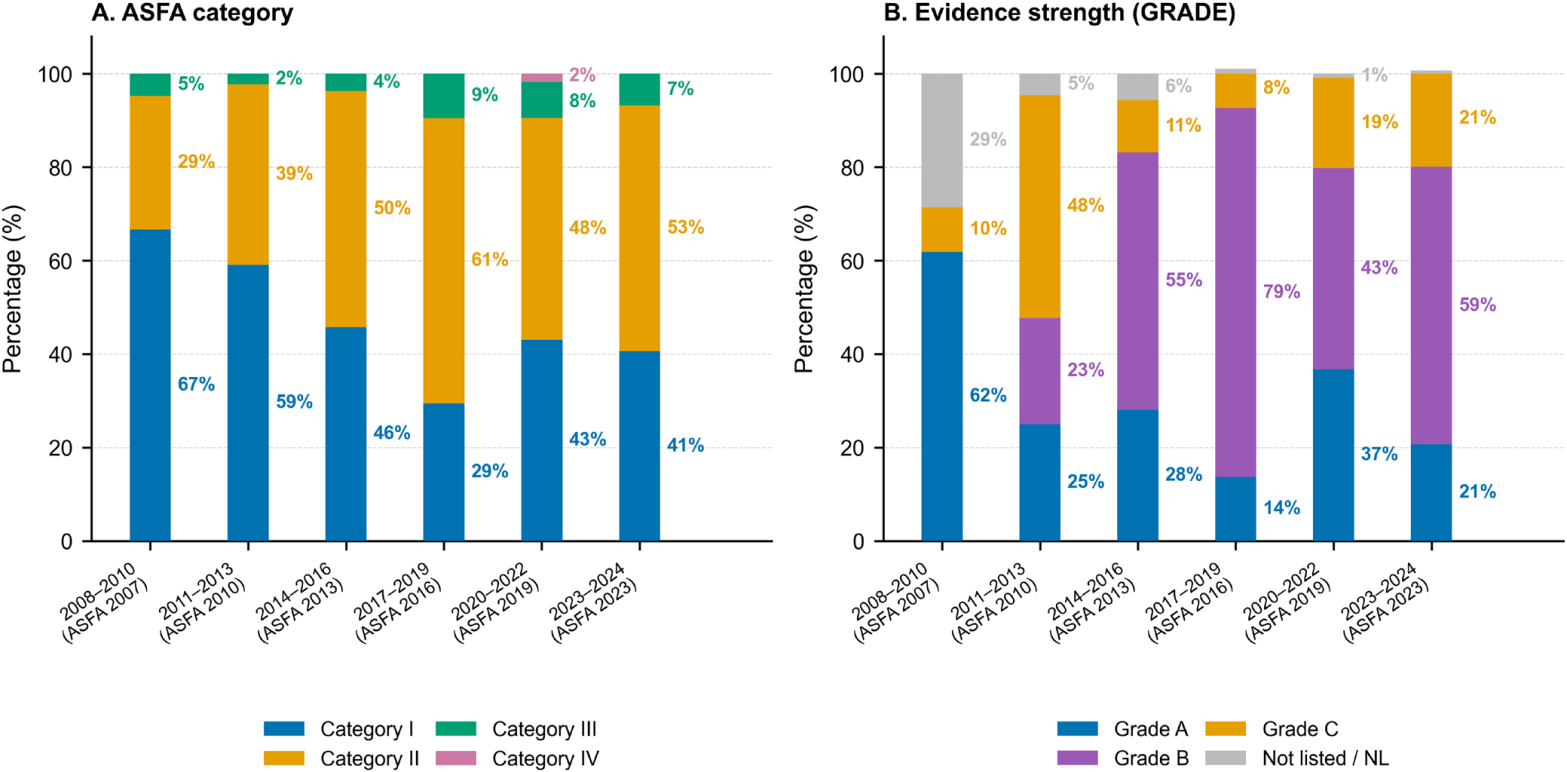
Distribution of ASFA categories (Panel A) and evidence strength by GRADE level (Panel B) per triennium (2008–2024). Each triennium is labeled with the corresponding ASFA guideline edition. ASFA, American Society for Apheresis; GRADE, Grading of Recommendations Assessment, Development and Evaluation; NL, not listed.

Regarding classification according to successive ASFA guideline editions, categories I and II predominated throughout the study period, with a relative decrease in category III and minimal representation of category IV. Among conditions with the greatest variation, ANCA‐associated vasculitis alternated between categories I and III across triennia, with a notable decline in indications for PE after 2023, following its reclassification to category III. Most other indications remained stable; however, variations were observed in acute antibody‐mediated renal rejection and genetically determined thrombotic microangiopathies (Figure 6).

## 4 Discussion

This study describes the clinical trends in PE over a 16‐year period across four high‐complexity referral institutions in Bogotá, representing one of the largest multi‐center cohorts reported in Latin America. Our findings demonstrate a progressive epidemiological transition, where systemic autoimmune diseases have been gradually superseded by immune‐mediated neuroimmunological disorders as the primary indication for PE.

This therapeutic shift, which became particularly pronounced after 2014, directly reflects the expanded clinical availability of highly specific diagnostic biomarkers, such as anti‐aquaporin‐4 (AQP4‐IgG) and anti‐myelin oligodendrocyte glycoprotein (MOG‐IgG) antibodies [20]. Enriched diagnostic screening has enabled earlier, more precise differentiation of neuromyelitis optica spectrum disorders (NMOSD) and MOG antibody‐associated disease (MOGAD) from classical multiple sclerosis [21]. Concurrently, PE has become firmly established as a mandatory second‐line intervention for steroid‐refractory neuroimmunological relapses [22]. This practice is supported by recent clinical trial data and meta‐analyses demonstrating acute improvements in Expanded Disability Status Scale scores and visual acuity, with therapeutic benefits sustained up to 6–12 months [23]. Together, these diagnostic and therapeutic advances have driven the sustained expansion of neuroimmunological indications observed in our cohort.

Beyond changes in the indication profile, our data reflect an exponential increase in the absolute utilization of PE, escalating from 21 procedures in the 2008–2010 triennium to 266 in 2023–2024. This growth parallels the global expansion of apheresis practice and the progressive broadening of indications recognized by the American Society for Apheresis (ASFA), which expanded from 61 conditions in its early iterations to 166 in the 2023 guidelines [6]. In our specific healthcare setting, this surge represents a convergence of improved diagnostic infrastructure, growing clinician familiarity with apheresis techniques, and the distinct operational feasibility of membrane‐based PE. Unlike centrifugation devices, membrane‐based platforms can be seamlessly managed by nephrology teams in general wards and emergency departments without requiring intensive care unit (ICU) resources. Although a fraction of this increase may be attributable to the transition toward comprehensive electronic medical records in recent years, the sheer magnitude of the trend confirms a genuine rise in clinical demand. This reality underscores the urgent need for structured institutional resource planning and the establishment of a centralized national apheresis registry.

When compared with studies from Europe and Asia, it is noteworthy that hematological conditions constitute a substantial proportion of PE indications in European registries [24, 25, 26], whereas in single‐center series from India, renal indications predominate [27, 28]. This pattern is also reflected in our own regional experience: in a single‐center Colombian series, renal indications were the most frequent [29], consistent with the trend observed in other developing‐country settings where institutional case‐mix and referral patterns—rather than established international guidelines alone—appear to shape the predominant indication profile [30]. These differences may be partly explained by the technology employed: centrifugation‐based apheresis predominates in Europe and in other single‐center series using discontinuous‐flow centrifugation [30], while membrane‐based techniques (used in our institutions) account for only 0.8% of procedures in those registries [26]. Given that these modalities differ in separation efficiency, operational costs, and hospital availability, it is plausible that the predominant technology inherently shapes institutional preferences and local indication profiles.

Relevant changes in ASFA classification were also observed during the study period, particularly for ANCA‐associated vasculitis and genetically determined thrombotic microangiopathies [5, 6]. These changes were most evident in ANCA‐associated vasculitis, where reclassification to category III was accompanied by a marked decline in the use of PE after 2023. This trend reflects the evolution of scientific evidence: early studies such as MEPEX [31] suggested the benefit of PE in severe renal disease, whereas the more robust PEXIVAS trial [32] failed to demonstrate reductions in mortality or progression to end‐stage kidney disease. Following these results, the use of PE in rapidly progressive glomerulonephritis declined substantially. Nevertheless, debate persists in critical scenarios such as diffuse alveolar hemorrhage, where evidence remains limited and clinical practice varies across institutions [33, 34].

Regarding clinical outcomes, the improvement rate observed in neuroimmunological disorders (78.2%) is consistent with international series reporting response rates ranging from 64.5% to 85% [35, 36, 37, 38]. This pattern may be explained by predominantly antibody‐mediated pathophysiology, which is amenable to rapid removal by PE. Indeed, some studies have shown that earlier initiation of therapy is associated with a higher likelihood of complete recovery in neuromyelitis optica spectrum disorders [37]. In contrast, the more limited response observed in systemic autoimmune diseases likely reflects more complex, multisystem inflammatory mechanisms that are less dependent on the plasma compartment, thereby reducing the immediate impact of PE on established tissue inflammation [39].

Mortality in our cohort was concentrated among systemic autoimmune and hematological conditions. Although neurological disorders represented the majority of indications, their in‐hospital mortality was low (4.2%), whereas rheumatological and hematological indications carried substantially higher in‐group mortality (25.4% and 25.0%, respectively). At the individual diagnosis level, catastrophic antiphospholipid syndrome (40.0%), systemic lupus erythematosus (25.0%), ANCA‐associated vasculitis (23.7%), and thrombotic microangiopathy (23.7%) showed the highest case‐fatality rates, consistent with the fulminant, multiorgan nature of these conditions rather than the technical failure of PE itself [40]. The leading causes of death were progression of the underlying disease (46.4%) and infectious complications (30.4%), whereas no death was attributable to a direct complication of the procedure. The predominance of disease progression reinforces that mortality was driven primarily by disease severity; at the same time, the substantial contribution of infection underscores the vulnerability of these patients, in whom concurrent immunosuppressive therapy and the removal of immunoglobulins by PE may heighten the risk of infection [40, 41].

Notably, 31.9% of deaths occurred during active treatment—on the same day as the last session (13.0%) or within 48 h of the last session in patients who had not completed a standard five‐session cycle (23.2%)—indicating that a substantial proportion of patients reached PE in an already critical, often irreversible clinical state. This pattern suggests that in severe systemic autoimmune disease, the benefit of PE may be constrained by delayed initiation and advanced organ damage and highlights the need to evaluate earlier referral and treatment thresholds in future prospective studies [42, 43, 44].

Despite the high early mortality associated with specific conditions, overall in‐hospital mortality declined substantially over the study period, decreasing from 28.6% in 2008–2010 to 6.0% in 2023–2024. This decline largely parallels the progressive shift in indications toward lower‐mortality neuroimmunological disorders and away from high‐mortality systemic autoimmune conditions, but probably also reflects earlier diagnosis, broader access to PE, and accumulated institutional experience [28]. A transient increase in mortality during the 2020–2022 triennium (10.3%) coincided with the COVID‐19 pandemic, a period marked by delayed presentations, healthcare system strain, and a higher proportion of critically ill patients [45]. Although our design does not allow causal attribution, this finding is consistent with the reported impact of the pandemic on outcomes of patients with immune‐mediated diseases and merits further analysis.

Our cohort shows a high proportion of patients managed in general wards and emergency departments, a trend particularly evident after 2012 and mainly observed in neurological and renal conditions. This distribution contrasts with regional and international studies, where filtration‐based PE is predominantly performed in intensive care units [8, 46]. Despite this less invasive care setting, the complication rate in our series was higher than that reported in European registries, where rates range from 3% to 7% [27]. While hypotension is the most common complication in those registries [46], electrolyte disturbances (particularly hypocalcemia) predominated in our cohort. This difference may be related to variations in calcium or magnesium replacement protocols, which are not always detailed in international reports. Systemic anticoagulation was rarely used (2.1% of sessions), and no episodes of circuit clotting were documented, consistent with reports from other cohorts in which routine anticoagulation was also omitted [11, 12, 29]. This contrasts with registries where approximately 60% of sessions require anticoagulation [46, 47], highlighting our specific experience with membrane‐based techniques.

Analytical evaluation of our data highlights several institutional strengths, including a multicenter design, a long observation period, consecutive inclusion of all treated patients, and systematic use of ICD‐10 coding and ASFA categorization. The cohort size allowed precise characterization of temporal trends and assessment of the real‐world impact of ASFA guideline changes on clinical practice. Additionally, the use of REDCap and standardized data collection procedures, as outlined in an investigator's manual, enhanced methodological rigor [17].

Certain limitations inherent to the retrospective design must be acknowledged, including reliance on the completeness and accuracy of clinical records. Clinical outcomes were limited to the index hospitalization during which PE was performed, precluding the assessment of long‐term outcomes, relapses, and delayed complications. Moreover, because the cohort included a wide range of indications, clinical outcomes were assessed using disease‐specific criteria and assessment scales based on the treating specialist's evaluation, rather than a standardized outcome measure across the entire cohort. As a result, some variability in outcome assessment may have occurred, particularly among patients with neurological disorders. Nevertheless, the large sample size, extended study period, and inclusion of a broad spectrum of indications provide a comprehensive overview of real‐world PE practice in a high‐complexity referral center.

## 5 Conclusion

This study documents a profound shift in PE indications over a 16‐year period in Colombia, characterized by a steady transition away from systemic autoimmune diseases toward immune‐mediated neuroimmunological disorders, catalyzed by the regional availability of specific AQP4‐IgG and MOG‐IgG serological testing. Furthermore, real‐world clinical practice demonstrated immediate compliance with international evidence and ASFA guideline updates, as evidenced by the rapid decline in PE utilization for ANCA‐associated vasculitis following its reclassification to Category III. Future prospective studies are warranted to evaluate long‐term functional outcomes, optimal timing of therapy initiation, and formal cost‐effectiveness ratios for membrane‐based technologies in Latin America, alongside the creation of a national apheresis registry to ensure continuous epidemiological surveillance.

## Funding

The authors have nothing to report.

## Ethics Statement

The protocol was approved by the Institutional Review Boards and Ethics Committees of all participating institutions: Hospital Universitario San Ignacio/Pontificia Universidad Javeriana (codes FM‐CIE‐1408‐24 and 2024/289), Hospital Universitario Nacional de Colombia (code CEI‐2024‐12‐07), Fundación Universitaria Sanitas (code CEIFUS 866‐25), and Hospital Universitario Clínica San Rafael (code CEI 054‐2025).

## Consent

For this retrospective analysis of historical data spanning from 2008 to 2024, the Institutional Review Board waived the requirement for written informed consent as all data were de‐identified and anonymized to ensure patient confidentiality.

## Conflicts of Interest

The authors declare no conflicts of interest.

## Supporting information


**Table S1:** Temporal trends in plasma exchange indications and their classification according to ASFA guidelines.

## Data Availability

The data that support the findings of this study are available from the corresponding author upon reasonable request. Patient‐level data are not publicly available due to privacy regulations and ethics committee restrictions.
