# Supplementary material for: Trends in Clinical Indications for Plasma Exchange in Adult Patients Between 2008 and 2024 in Bogotá, Colombia
Source: J Clin Apher. 2026 Jul 28;41(4):e70161. doi: 10.1002/jca.70161 (PMC13414631; doi:10.1002/jca.70161)
Supplement: Supplementary file 1 — Table S1: Temporal trends in plasma exchange indications and their classification according to ASFA guidelines. [file JCA-41-e70161-s001.docx]

**Supplementary Table S1. Temporal trends in plasma exchange indications and their classification according to ASFA guidelines.**

| **Most frequent conditions** | **Cases, n (%)** | **ASFA category** |
| --- | --- | --- |
| **2008–2010 (N = 21) — ASFA 2007** | | |
| Myasthenia gravis | 6 (28.6%) | Category I |
| ANCA-associated vasculitis | 3 (14.3%) | Category I |
| Thrombotic microangiopathy | 2 (9.5%) | Category I |
| Catastrophic antiphospholipid syndrome | 2 (9.5%) | Category II |
| Cardiac allograft rejection | 2 (9.5%) | Category II |
| Renal allograft rejection | 1 (4.8%) | Category II |
| Post-transplant FSGS | 1 (4.8%) | Category I |
| Acute encephalitis / ADEM | 1 (4.8%) | Category II |
| Hypertriglyceridemic pancreatitis | 1 (4.8%) | Category III |
| Anti-GBM disease | 1 (4.8%) | Category I |
| **2011–2013 (N = 44) — ASFA 2010** | | |
| ANCA-associated vasculitis | 9 (20.5%) | Category I |
| Myasthenia gravis | 7 (15.9%) | Category I |
| Renal allograft rejection | 6 (13.6%) | Category I |
| Optic neuritis / NMOSD | 5 (11.4%) | Category II |
| Systemic lupus erythematosus | 5 (11.4%) | Category II |
| Catastrophic antiphospholipid syndrome | 3 (6.8%) | Category II |
| Cryoglobulinemia | 2 (4.5%) | Category I |
| Acute encephalitis / ADEM | 2 (4.5%) | Category I |
| Other neuropathy | 1 (2.3%) | Category II |
| Thrombotic microangiopathy | 1 (2.3%) | Category I |
| **2014–2016 (N = 107) — ASFA 2013** | | |
| Optic neuritis / NMOSD | 41 (38.3%) | Category II |
| ANCA-associated vasculitis | 23 (21.5%) | Category I |
| Myasthenia gravis | 13 (12.1%) | Category I |
| Systemic lupus erythematosus | 6 (5.6%) | Category II |
| CIDP | 4 (3.7%) | Category I |
| Acute encephalitis / ADEM | 4 (3.7%) | Category II |
| Guillain–Barré syndrome | 3 (2.8%) | Category I |
| Thrombotic microangiopathy | 3 (2.8%) | Category I |
| Cryoglobulinemia | 2 (1.9%) | Category I |
| Paraproteinemia / hyperviscosity | 2 (1.9%) | Category I |
| **2017–2019 (N = 95) — ASFA 2016** | | |
| Optic neuritis / NMOSD | 50 (52.6%) | Category II |
| Myasthenia gravis | 19 (20%) | Category I |
| ANCA-associated vasculitis | 9 (9.5%) | Category III |
| Thrombotic microangiopathy | 4 (4.2%) | Category III |
| Renal allograft rejection | 4 (4.2%) | Category I |
| Systemic lupus erythematosus | 4 (4.2%) | Category II |
| Guillain–Barré syndrome | 2 (2.1%) | Category I |
| Multiple sclerosis | 1 (1.1%) | Category II |
| Autoimmune hemolytic anemia | 1 (1.1%) | Category II |
| Catastrophic antiphospholipid syndrome | 1 (1.1%) | Category II |
| **2020–2022 (N = 223) — ASFA 2019** | | |
| Optic neuritis / NMOSD | 67 (30%) | Category II |
| Guillain–Barré syndrome | 38 (17%) | Category I |
| Thrombotic microangiopathy | 21 (9.4%) | Category I |
| Acute encephalitis / ADEM | 18 (8.1%) | Category II |
| ANCA-associated vasculitis | 17 (7.6%) | Category I |
| Systemic lupus erythematosus | 17 (7.6%) | Category II |
| Myasthenia gravis | 10 (4.5%) | Category I |
| Multiple sclerosis | 9 (4%) | Category II |
| CIDP | 7 (3.1%) | Category I |
| Renal allograft rejection | 6 (2.7%) | Category I |
| **2023–2024 (N = 266) — ASFA 2023** | | |
| Optic neuritis / NMOSD | 108 (40.6%) | Category II |
| Acute encephalitis / ADEM | 26 (9.8%) | Category I |
| Guillain–Barré syndrome | 21 (7.9%) | Category I |
| Renal allograft rejection | 20 (7.5%) | Category I |
| Myasthenia gravis | 16 (6%) | Category I |
| ANCA-associated vasculitis | 15 (5.6%) | Category III |
| Multiple sclerosis | 13 (4.9%) | Category II |
| Thrombotic microangiopathy | 9 (3.4%) | Category I |
| Systemic lupus erythematosus | 8 (3%) | Category II |
| Graves disease / hyperthyroidism | 8 (3%) | Category II |

*Triennia were assigned by the date of the first plasma exchange session. The ten most frequent indications are shown per triennium; percentages are relative to the total number of patients in each triennium. ASFA category reflects the modal classification of each condition under the guideline edition in force during that triennium. ADEM, acute disseminated encephalomyelitis; ANCA, antineutrophil cytoplasmic antibody; ASFA, American Society for Apheresis; CIDP, chronic inflammatory demyelinating polyradiculoneuropathy; FSGS, focal segmental glomerulosclerosis; GBM, glomerular basement membrane; NMOSD, neuromyelitis optica spectrum disorder.*
